# Functional Outcome Based on Mechanical Axis Alignment Following Total Knee Arthroplasty

**DOI:** 10.7759/cureus.22553

**Published:** 2022-02-24

**Authors:** Himanshu Charaya, Harpreet S Gill, Rahul Bhan

**Affiliations:** 1 Department of Orthopedics, Satguru Partap Singh (SPS) Hospital, Ludhiana, IND

**Keywords:** knee society score, functional outcome, long-leg radiographs, alignment, mechanical axis, total knee arthroplasty

## Abstract

Background

Total knee arthroplasty (TKA) is the most commonly performed and highly successful surgical intervention for end-stage osteoarthritis of the knee, and it offers patients pain relief, functional recovery, and improved quality of life. The success of knee arthroplasty depends on various factors such as precise surgical technique, alignment of the limb and components, patient selection, and compliance with rehabilitation. Mechanical alignment of the lower limb has been considered an important factor in planning and assessing the success of TKA. Optimal alignment remains a matter of controversy; hence, it is paramount to assess the alignment and functional outcomes.

Aim and objective

This study aimed to evaluate the reliability of conventional instrumentation in imparting the intended femoral and tibial coronal alignment, as well as study the functional outcome among the neutrally aligned outliers with respect to the mechanical axis of the lower limb using standing long-leg radiographs.

Methodology

This is a prospective, hospital-based, observational study that was conducted on 60 knees in 42 patients with primary osteoarthritis of the knee joint in the department of orthopedics, Satguru Partap Singh (SPS) Hospitals, Ludhiana. Patients undergoing total knee arthroplasty who fulfilled the inclusion criteria were included in our study and evaluated using Knee Society Score and knee flexion range at periodic follow-up till six months. Preoperative and postoperative standing long-leg radiographs were done for all the patients, and their functional outcome was compared among inliers and outliers.

Results

Out of these 60 total knee arthroplasties, 18 patients were operated on both knees, and 24 patients were operated on a single knee. There were 25 female patients and 17 male patients. The mean for pre-operative mechanical axis alignment angle was 11.88° ± 5.63° with a range from -3° to 27°, which changed to 2.90° ± 1.59° with a range from 0° to 8° at six months follow-up. It was observed that 42 of the knees were in the inliers, and the remaining 18 knees were in the outliers group. On comparison among inliers and outliers, we found that the mean range of motion was 108.29° ± 4.82° for the inliers group and 106.11° ± 4.04° for the outliers group (p = 0.091), depicting non-significant statistical comparison. Mean Knee Society Score values in inliers and outliers group were 152.45 ± 5.33 and 151.61 ± 3.55, respectively (p = 0.740), showing no statistical significance.

Conclusion

At six months follow-up, there is no difference in the knee range of motion and Knee Society Scores between mechanical axis inliers and outliers. Thus, we conclude that although every knee arthroplasty is intended to have neutral mechanical alignment, there is no effect of mild mechanical axis malalignment on functional outcome following total knee arthroplasty in the short term.

## Introduction

Osteoarthritis (OA) of the knee is the most common articular disease worldwide and a leading cause of chronic disability, particularly in the geriatric population [1]. Total Knee Arthroplasty (TKA) is the most common and highly successful surgical intervention for end-stage OA, and it offers patients pain relief, functional recovery, and improved quality of life [2-4]. TKA is a bony and essentially a soft-tissue procedure where much attention has been given to the alignment of components, particularly in the coronal plane. Long-term outcome after TKA is mainly dependent on the neutral alignment of the knee, which reduces the mechanical and shear stresses on the bearing surfaces and the bone-prosthesis interface. Furthermore, neutral alignment aids in balancing the forces transmitted through the soft tissue envelope of the knee joint [5]. This choice of alignment is based on the long-held belief that postoperative alignment of the lower limb should be within ±3° of the neutral mechanical axis [6]. Restoration of this neutral mechanical alignment of the knee is one of the key criteria of a successful TKA [7]. Alignment is almost exclusively measured using long-leg radiographs (LLR), especially when cited to evaluate clinical and functional outcomes [8]. To attain this goal, various alignment strategies and surgical techniques have been used by surgeons like conventional TKA, computer navigated/assisted surgery, and patient-specific guides. The classic axiom in TKA surgery is that the Hip-Knee-Ankle angle needs to be 180°±3° and that the survival and function are directly related to its alignment [9]. Some authors have shown better functional outcomes amongst those with alignment within the ±3° range [10-11], while others have emphasized that improved alignment is not necessarily associated with improved function [12-13]. As optimal alignment remains a matter of controversy and the subsequent emergence of computer navigation, patient-specific cutting blocks and the concept of Kinematic alignment have further fueled this never-ending debate; thus, we found it paramount to assess the outcome of conventional instrumentation among the mechanically neutrally aligned and malaligned knees.

## Materials and methods

**Figure 1:**
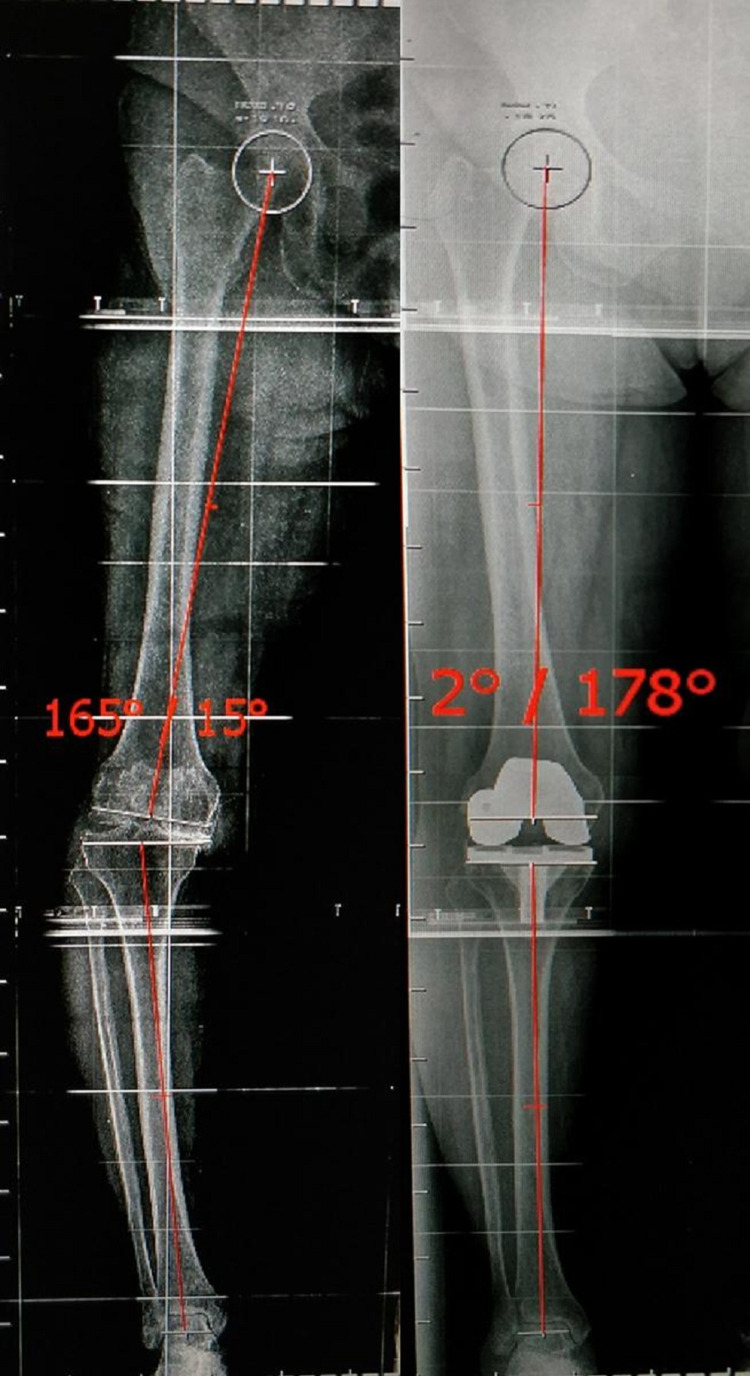
Long-leg radiograph showing measurement of the Tibio-femoral mechanical angle preoperative and postoperative for Case 3. As the angle has come out to be 2° and is within ±3° range, hence case 3 lies in the inliers group.

**Figure 2:**
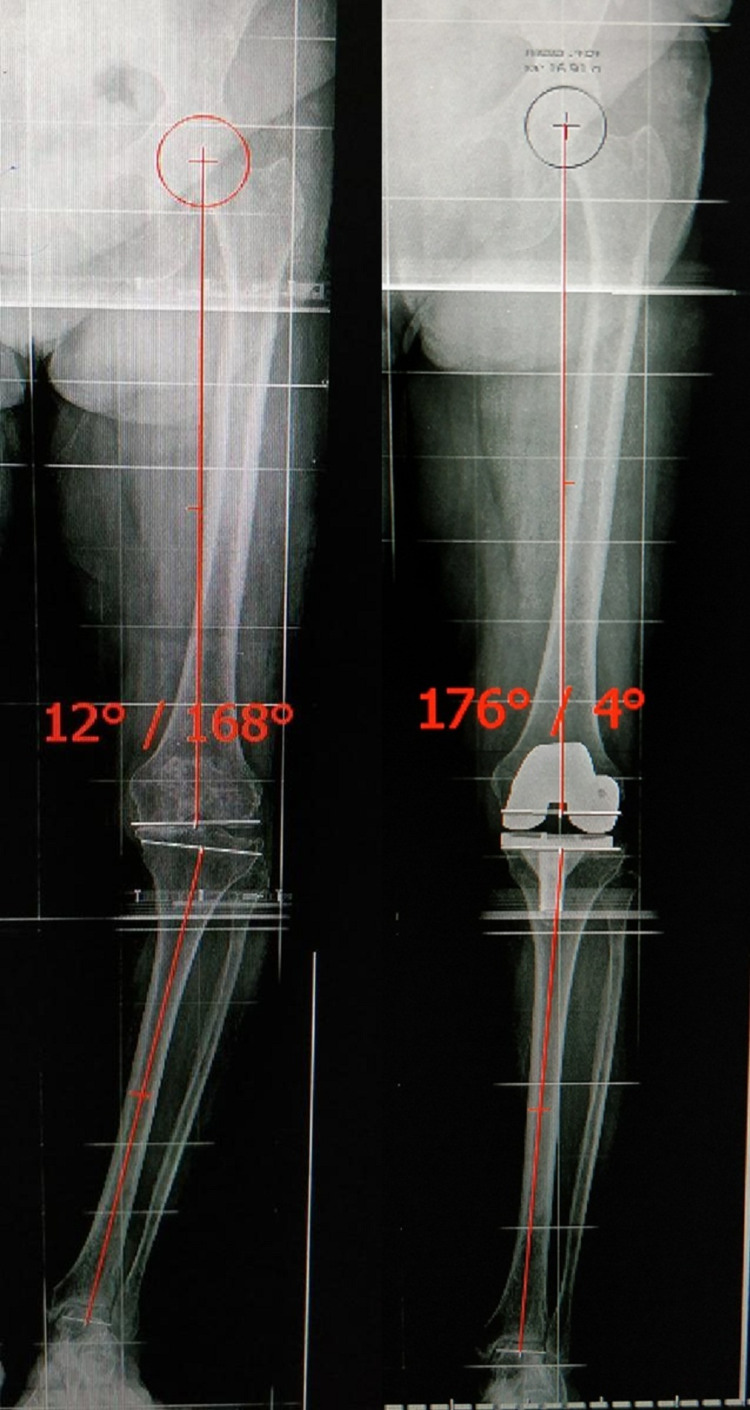
Long-leg radiograph showing measurement of the Tibio-femoral mechanical angle preoperative and postoperative for Case 2. As the angle comes outside of ±3° range, i.e., 4°, hence case 2 lies in the outliers group

**Figure 3:**
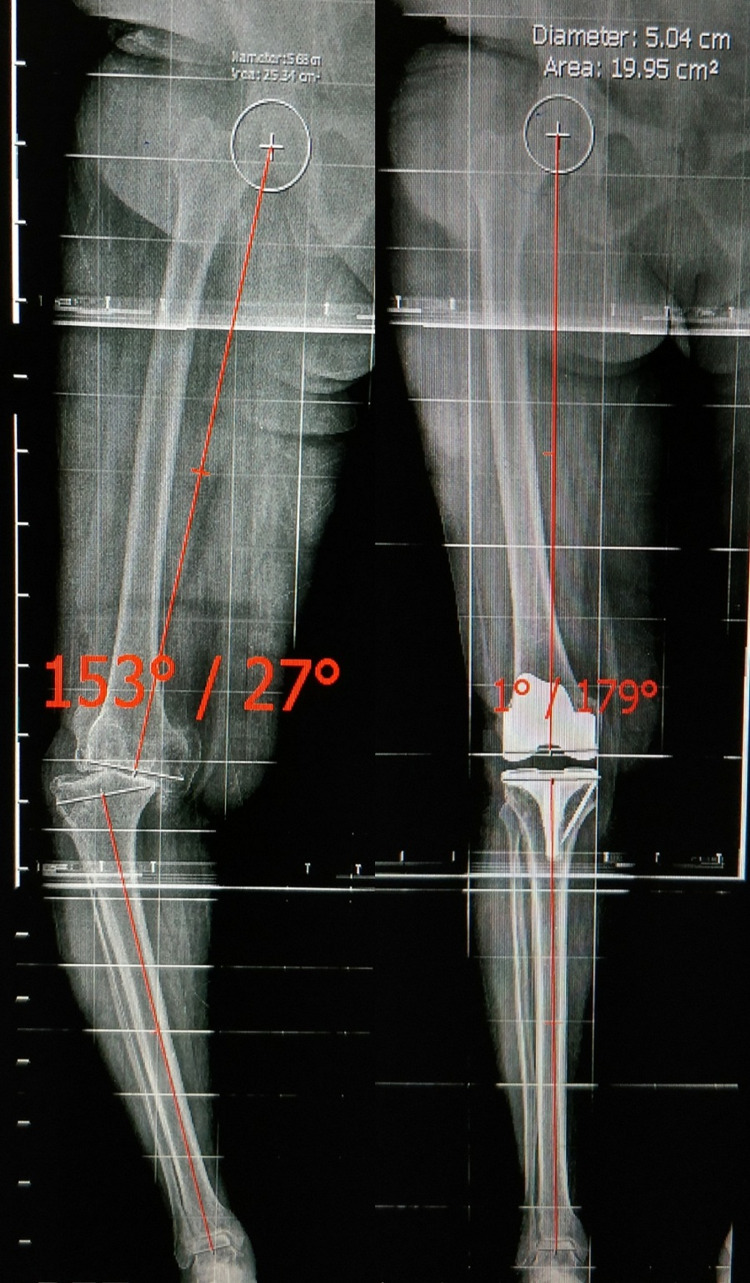
Long-leg radiograph showing measurement of the Tibio-femoral mechanical angle preoperative and postoperative for Case 1. As the angle comes within ±3°, hence case 1 lies in the inliers group.

This was a prospective, hospital-based, observational study that was conducted on 60 knees with primary OA of the knee joint from October 2016 to June 2017 in the department of Orthopaedics, SPS Hospitals, Ludhiana. A total of 84 cases of TKAs were performed in the period between October 2016 and June 2017. Six knees in six patients with rheumatoid arthritis, five patients who did not sign written consent for the study, eight cases lost in follow-up, and five patients with the malunited distal femur and proximal tibia fractures were excluded from the study. Sixty cases undergoing TKA who fulfilled the inclusion criteria were included in our study. Preoperatively clinical examination was done, and Knee Society Score values and knee range of motion using the goniometer were measured and documented. The radiographic evaluation consisted of standard anteroposterior, lateral, and standing long-leg radiographs/X-ray scanogram as per the technique by Paley et al. [14]. All surgeries were performed by two senior surgeons in the laminar flow operating rooms. The standard anterior midline skin incision with a medial parapatellar approach was used in all surgeries. Intra-operatively, the proximal tibia cut was made perpendicular to the mechanical axis of the tibia, using an external tibial alignment system attached to the proximal tibia and the ankle. Rotation of the tibial component was referenced from the medial third of the tibial tuberosity. The flexion tensioning jig was placed into the flexion gap, tensioning the gap, and was positioned parallel to the trans-epicondylar axis. Then anterior and posterior femoral resections were performed. After accurate balancing in flexion, the tensioning jig was placed into the extension gap with an intramedullary guide. All surgeries were carried out by the gap balancing technique. Prophylactic antibiotics were given preoperatively and postoperatively for two days, DVT prophylaxis and post-operative rehabilitation were similar in all the cases. Intravenous analgesia/patient-controlled anaesthesia/continuous femoral nerve block anaesthesia/epidural anaesthesia was administered for postoperative pain control. Postoperatively patients were examined at two weeks, six weeks, three months, and six months of follow-up, Knee Society Score [15], knee flexion range were calculated and documented at each visit. An X-Ray scanogram of the concerned limb was obtained at six months follow-up. The mechanical axis of the lower extremity was determined by drawing a line from the centre of the femoral head to the centre of the ankle joint, Maquet’s line, which corresponds to an approximately 3° slope compared with that of the vertical axis. This was further subdivided into the femoral mechanical axis, which runs from the head of the femur to the intercondylar notch of the distal femur in preoperative evaluation and post-TKA cases from the centre of the femoral head to the centre of the width of the distal femoral prosthesis, and the tibial mechanical axis, which extends from the centre of the proximal tibia or as in post-TKA cases from the centre of the width of the tibial prosthesis to the centre of the ankle. The tibiofemoral mechanical angle/mechanical axis angle of the lower limb was calculated and documented as shown in Figures 1-3. When in varus, it was documented as positive in degrees, and while in valgus, it was documented as negative. Patients were divided into two groups based on mechanical alignment as neutral/inliers group with less than ±3° [group 1] and outliers with axis deviation more than ±3° [group 2].

Statistical analysis was performed using the IBM Corp. Released 2012. IBM SPSS Statistics for Windows, Version 21.0. Armonk, NY: IBM Corp. All values were expressed as mean ± standard deviation (SD) and as the median. The values were evaluated using the Mann-Whitney U test, and a p-value<0.05 was considered to indicate statistical significance. 

## Results

**Figure 4:**
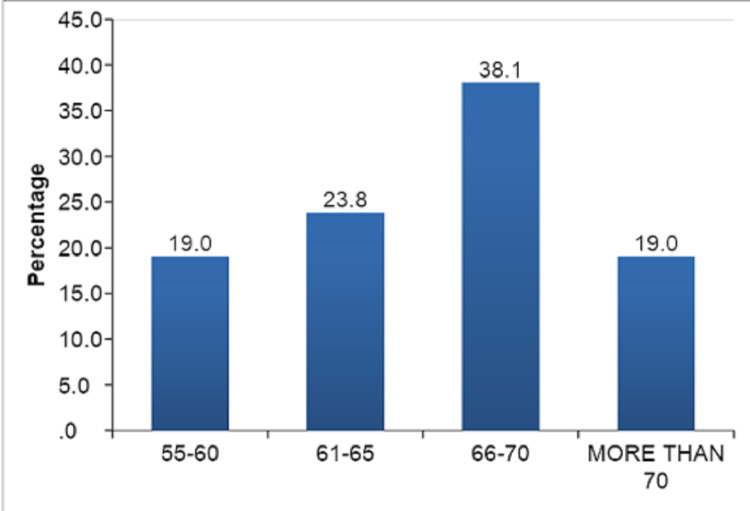
Age-wise distribution.

Out of these 60 TKAs, 18 patients were operated on for bilateral TKAs, and 24 patients were operated on for unilateral TKA. There were 25 female patients and 17 male patients. The mean age in our study was 65.98 ± 5.90 years, with a range from 55 to 81. Most patients in our study were from the 66-70 years age group. Age-wise distribution of patients in our study is shown in Table 1 and Figure 4.

**Table 1:** Age distribution in our study.

Age group (years)	No. of Patients	Percentage
55-60	8	19.0
61-65	10	23.8
66-70	16	38.1
>70	8	19.0
Total	42	100.0

**Figure 5:**
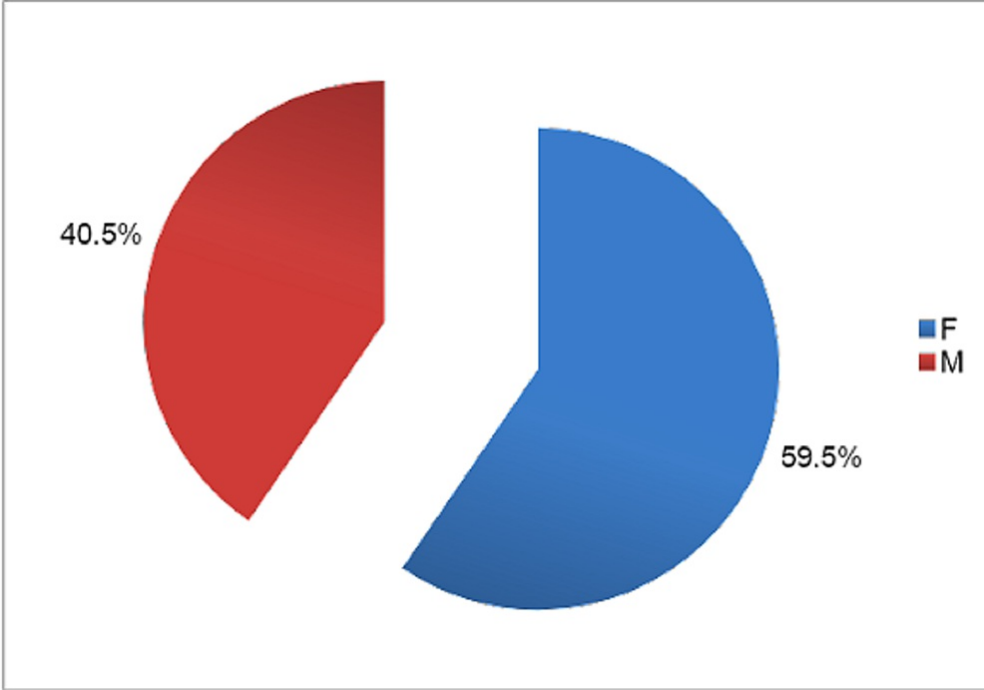
Sex distribution in our study.

There were 25 female patients and 17 male patients in our study. The majority of patients were females in our study. The sex-wise distribution of patients is shown in Figure 5.

Out of the 60 TKA cases, 27 were operated on for the left side and 33 for the right side. Side distribution is shown in Table 2.

**Table 2:** Side-wise distribution in our study.

Side	No. of Knees	Percentage
Left	27	45.0
Right	33	55.0
100.0	100.0	100.0

Upon measurement of the angle between the mechanical axis of the femur and the mechanical axis of the tibia, it was observed that 42 of the knees were inliers and the remaining 18 knees were in the outliers group. The distribution of patients among inliers and outliers groups is shown in Table 3.

**Table 3:** Distribution based on mechanical axis alignment.

Inlier/Outlier	No. of knees	Percentage
Inliers {Group 1}	42	70.0
Outliers {Group2}	18	30.0
Total	60	100.0

**Figure 6:**
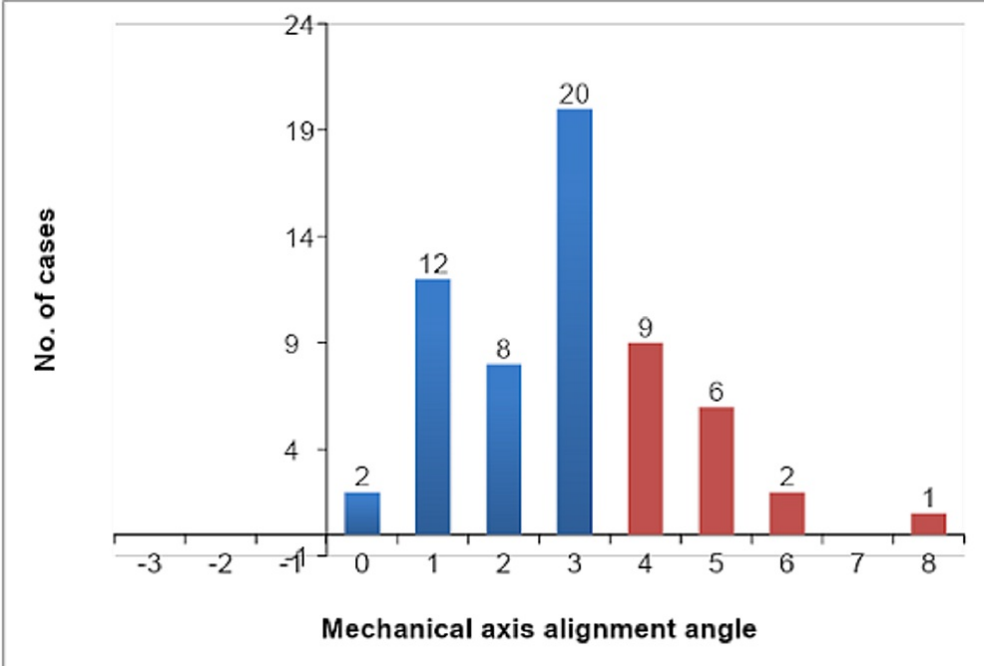
Mechanical axis-wise distribution of cases.

The mean for preop mechanical axis alignment angle was 11.88° ± 5.63° with a range from -3° to 27°, which got changed to 2.90° ± 1.59° with a range from 0° to 8° degrees at six months of follow up after TKA. Most of our cases had an angle of 3° between the mechanical axis of the femur and the mechanical axis of the tibia. None of our cases had valgus alignment postoperatively. The distribution of cases based on the mechanical axis angle of the lower limb is depicted in Figure 6.

**Figure 7:**
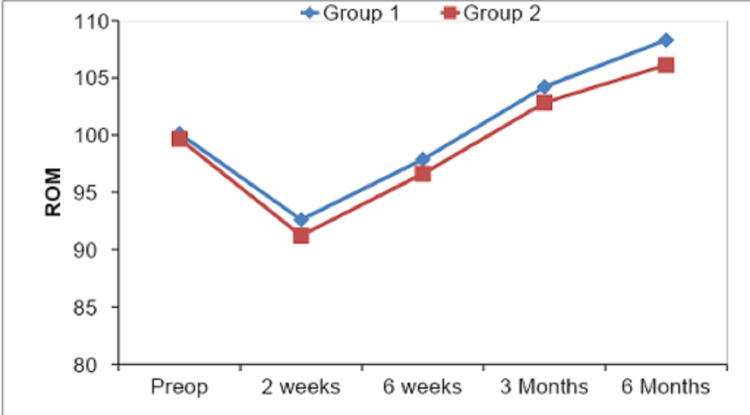
Graphical representation of mean ROM in degrees at each follow-up for inliers and outliers.

At the end of six months, the mean range of motion (ROM) for the inliers group of patients was 108.29° ± 4.82°, and for the outliers group was 106.11° ± 4.04°, which was not statistically significant and is represented in Table 4 and Figure 7.

**Table 4:** Comparison of mean range of motion between inliers and outliers.

ROM	Group 1 [Inliers] (N=42)		Group2 [Outliers] (N=18)		Z	p-value
	Mean	SD	Mean	SD		
Preop	100.12	9.08	99.67	5.35	-0.399	0.690
2 weeks	92.60	5.22	91.22	5.06	-0.510	0.610
6 weeks	97.86	4.40	96.61	3.73	-0.784	0.433
3 Months	104.21	5.13	102.83	2.57	-0.632	0.527
6 Months	108.29	4.82	106.11	4.04	-1.690	0.091

The mean for preop total Knee Society Score (KSS) was 87.78 ± 8.81, which got changed to 102.90 ± 5.88 at two weeks follow up and got further increased to 114.45 ± 5.22 at six weeks follow up and to 133.80 ± 5.72 at three months of follow-up. At six months, the mean KSS was calculated as 152.20 ± 4.85. Mean total KSS values at each follow-up are shown in Table 5.

**Table 5:** Showing mean Total KSS values at each follow-up.

Total Knee Society Score	N	Minimum	Maximum	Mean	SD
Preop	60	64	101	87.78	8.81
2 Weeks	60	81	117	102.90	5.88
6 Weeks	60	100	128	114.45	5.22
3 Months	60	124	148	133.80	5.72
6 Months	60	140	165	152.20	4.85

**Figure 8:**
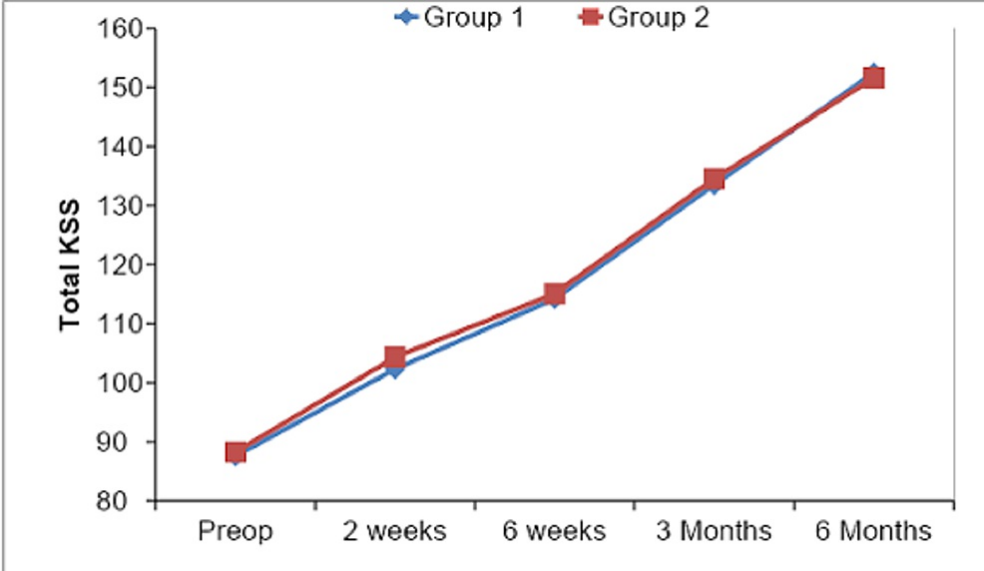
Graphical representation of comparison of KSS values between inliers and outliers at each follow-up.

The correlation of total Knee Society Score and mechanical axis inliers and outliers is shown in Table 6 and Figure 8. At six months mean KSS for inliers was 152.45 ± 5.33 as compared to 151.61 ± 3.55 among outliers.

**Table 6:** Comparing mean KSS values between inliers and outliers at each follow-up.

Total Knee Society Score	Group 1 [Inliers] (N=42)		Group 2 [Outliers] (N=18)		Z	p-value
	Mean	SD	Mean	SD		
Preop	87.57	9.42	88.28	7.43	-0.048	0.961
2 weeks	102.29	4.85	104.33	7.75	-1.873	0.061
6 weeks	114.19	5.04	115.06	5.72	-0.210	0.833
3 Months	133.50	6.09	134.50	4.83	-0.987	0.323
6 Months	152.45	5.33	151.61	3.55	-0.380	0.704

**Figure 9:**
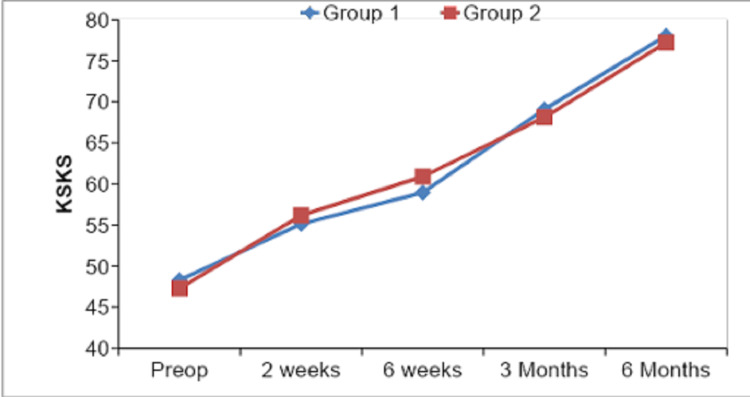
Graphical representation of comparison of mean Knee Society Knee Score values for inliers and outliers.

The correlation of Knee Society Knee Score and mechanical axis inliers and outliers is shown in Table 7 and Figure 9. At six months of follow-up, the mean value for KS in the inliers group was 78.05 ± 3.36 as compared to 77.22 ± 3.89 in the outliers group, which was not statistically significant.

**Table 7:** Showing comparison of mean KS values of inliers and outliers at each follow-up.

Knee Society Knee Score	Group 1 [Inliers] (N=42)		Group2 [Outliers] (N=18)		Z	p-value	
	Mean	SD	Mean	SD			
Preop	48.29	7.21	47.28	7.50	-0.501	0.616	
2 weeks	55.12	4.26	56.17	4.93	-1.693		0.090
6 weeks	58.95	3.80	60.89	3.32	-1.840	0.066	
3 Months	69.07	3.10	68.11	4.57	-0.926	0.355	
6 Months	78.05	3.36	77.22	3.89	-0.891	0.373	

**Figure 10:**
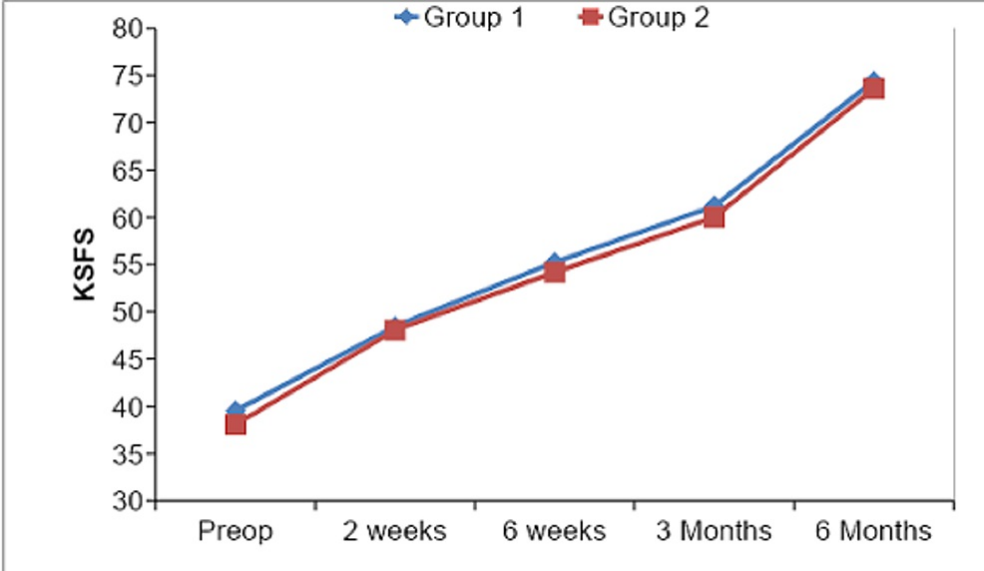
Graphical representation of comparison of Knee Society Function sub-score between inliers and outliers.

The mean for preop function score (FS) component of total Knee Society Score was 39.08 ± 4.91, which got changed to 48.33 ± 3.28 at two weeks of follow-up and further to 54.92 ± 2.98 at six weeks of follow-up and got further increased to 60.83 ± 3.08 at three months of follow-up. At six months of follow-up, the mean FS was calculated as 74.17 ± 3.70. Correlation of Knee Society Function score and mechanical axis inliers and outliers is shown in Table 8 and Figure 10. At six months, the mean value for FS in the inliers group was 74.40 ± 3.70 as compared to 73.61 ± 3.76 in the outliers group.

**Table 8:** Comparing FS between inliers and outliers.

Knee Society Function Score	Group1 [Inliers] (N=42)		Group 2 [Outliers] (N=18)		Z	p-value
	Mean	SD	Mean	SD		
Preop	39.52	4.92	38.06	4.89	-1.124	0.261
2 weeks	48.45	3.02	48.06	3.89	-0.493	0.622
6 weeks	55.24	2.69	54.17	3.54	-1.288	0.198
3 Months	61.19	2.66	60.00	3.83	-1.203	0.229
6 Months	74.40	3.70	73.61	3.76	0.564	0.525

## Discussion

TKA in end-stage OA knee patients in whom conservative methods of management have been exhausted has come out to have excellent results provided sound surgical technique and proper patient selection. Pain relief, restoration of normal limb alignment, and restoration of a functional range of motion are three main goals of TKA. Various factors such as lower limb alignment, rotational alignment of components, soft tissue balancing, and patella-femoral joint tracking have been linked to having an impact on clinical outcomes after TKA. John Insall, in 1985, had described the use of mechanical alignment in TKA and stated it to be better than anatomical alignment [16]. Obtaining a neutral mechanical alignment is traditionally regarded as an ideal target for primary TKA [6,10,16-19]. Mechanical alignment of the lower limb has been considered an important factor in planning and assessing the success of TKA [7]. Although primary TKA shows its effectiveness by reducing knee pain and increasing knee function in activities of daily living, the long-held axiom of neutral alignment, particularly in the coronal plane, is becoming more and more debatable, especially with the advent of kinematic alignment and the concept of constitutional varus [20]. Most of the studies that have addressed the impact of postoperative coronal alignment on the outcomes of TKA have had several limitations, including small sample size, old implant designs, short knee radiographs, and thus had not assessed overall limb alignment [6,8,18].

With respect to functional outcome, Matziolis et al. [13] had compared varus outliers with neutrally matched controls and suggested that functional outcome is not affected by residual postop varus alignment. Magnussen et al. [21] also compared neutrally aligned to residual varus group and found no difference in KSS or revision rates among the two groups. However, Vanlommel et al. [22] found superior functional scores in the mild residual varus patients. Results of these studies were contradictory to results by Manjunath et al. [23], Choong et al. [24], and Huang et al. [25], who had shown significantly better outcomes among neutrally aligned TKAs. Although axial alignment has been recognized as a critical factor in influencing the outcome of TKA and has been studied extensively in this regard but still is a matter of controversy. Our intention with this study was to find out the reliability of conventional instrumentation in imparting the intended femoral and tibial coronal alignment and to analyze functional outcomes among neutrally aligned knees and outliers for the mechanical axis of the lower limb.

The mean age of patients undergoing TKA in our study was 65.98 ± 5.90 years which was similar to Stulberg et al.'s [26] study with a mean age of 64.8 years, Decking et al.'s [27] study with a mean age of 67.3 ± 6.3 years, Gothesen et al.'s [28] study with a mean age of 67.7 ± 6.8 years, Slevin et al.'s [29] study with a mean age of 67.7 ± 9.9 years and Lutzner et al. [30] with a mean age of 69 years. 

The mean preop knee range of motion in our study was 99.98° ± 8.10° and postoperatively it was 92.18° ± 5.17° at two weeks, 97.48° ± 4.22° at six weeks, 103.8° ± 4.54° at three months, and 107.63° ± 4.67° at six months of follow-up. Gothesen et al.'s [28] and our values depict the same trends.

The Total Knee Society Score (KSS) in our study, its mean preop value was calculated as 87.78 ± 8.81, mean KSS postoperatively was 102.90 ± 5.88, 114.45 ± 5.22, 133.80 ± 5.72, and 152.20 ± 4.85 at two weeks, six weeks, three months and six months, respectively. As shown by Decking et al. [27] and Slevin et al. [29], our study depicted a similar increase in mean KSS value at sequential postoperative follow-ups. 

After observing the postoperative angle for MAA of the lower limb, we found that we could achieve the recommended 0° ± 3° of alignment in 42 of our TKA cases which corresponds to 70% of our cases, while the rest of our TKA cases, i.e., 18 cases had a deviation of more than ± 3° on standing long-leg radiographs at six months of follow-up. After critically reviewing the literature, the percentage of inliers from various studies, all of them having used conventional instrumentation for TKA, was collected and was found to be 80% in the study by Manjunath et al. [23], 62% in the study by Gothesen et al. [28], 61% in the study by Huang et al. [25], and our results were comparable to most of the studies. Furthermore, in our study, out of 18 cases of outliers, all were in varus, and as compared to 32% in the study by Gothesen et al. [28], none of our cases was a valgus outlier. 

On comparing our outcome parameters among inliers and outliers, we found that the mean ROM of group 1 (inliers) cases was 108.29° ± 4.82° and of group 2 (outliers) cases was 106.11° ± 4.04° with a p-value of 0.091 depicting a non-significant statistical comparison at the end of six months. Our mean KS values at six months postop follow-up were 78.05 ± 3.36, 77.22 ± 3.89 for group 1 and group 2, respectively, on comparison p-value came out to be 0.373, which showed no statistical significance. Mean FS values at six months were 74.40 ± 3.70, 73.61 ± 3.76 in group 1 and group 2, respectively, and their comparison showed a p-value of 0.525, thus stating a non-significant comparison. Similarly, mean KSS values in group 1 and group 2 were 152.45 ± 5.33 and 151.61 ± 3.55, respectively, with a p-value of 0.740 showing no statistical significance. Thus all the values of ROM, KS, FS, and total KSS at six months were insignificant among inliers and outliers, and our results were similar to other studies by Matziolis et al. [13] and Magnussen et al. [21]. Manjunath et al. [23] in their study had mean KS as 83.58 ± 7.59, 70 ± 24.49 among inliers and outliers with p-value 0.026, which was contrary to our study, and mean FS was 80.20 ± 15.42, 70 ± 24.49 among inliers and outliers respectively with p-value 0.209 which was similar to our study.

In our study, although there were no significant differences in clinical outcomes among inliers and outliers during the short-term follow-up, a further long-term follow-up will be necessary to make a firm conclusion. To achieve accurate mechanical axis alignment of 0° ± 3°, more ways have been employed, one of them is the use of computer navigation in TKA, which seems unlikely to be rewarded when compared to TKA done with traditional conventional instrumentation meticulously. Although describing alignment as a dichotomous variable is not acceptable to all but neutral, mechanical alignment will remain the gold standard, and accepting residual varus alignment without sufficient long-term data will be like going a step backward in surgical evolution.

It should be noted that we are aware of some inherent limitations. First, the follow-up period of our study was only six months which is less. Second, we have not taken the rotational alignment of femoral and tibial components into account, which can influence the results if considered. Third, alignment in the sagittal plane is not taken into account as posterior offset, and posterior tibial slope can affect ROM. Despite these limitations significance of the present study lies in the facts as it is a prospective observational study on a fairly large number of patients and followed up for six months, all patients were evaluated on weight-bearing long-leg radiographs, and there have been very fewer studies finding the reliability of conventional instrumentation in imparting intended mechanical alignment and further correlating outcomes among neutrally aligned or inliers and malaligned or outliers with respect to knee ROM and Knee Society scoring system.

## Conclusions

At six months follow-up, there is no difference in knee ROM, Knee Society Knee Score, Knee Society Function Score, and Total Knee Society Score between mechanical axis inliers and outliers. Thus, we concluded that although every TKA is intended to have neutral mechanical alignment, i.e., within ±3°, in the short term, there is no effect of mild mechanical axis malalignment on functional outcome following TKA. 

## Appendices

Appendix 1

Informed Consent Form

Subject identification number for this trial _________________________________

Title of the Project: ‘‘A study of post-operative mechanical axis alignment and it's functional outcome in total knee arthroplasty- a follow up study

Name of the Principal Investigator: Dr. Himanshu Tel. No. _________________

I have received the information sheet on the above study and have read and /or understood the written information.

I have been given the chance to discuss the study and ask questions.

I consent to take part in the study and I am aware that my participation is voluntary.

I understand that I may withdraw at my time without this affecting my future care.

I understand that the information collected about me from my participation in this research and sections of any of my medical notes may be looked at by responsible persons (ethics committee members/ regulatory authorities). I give access to these individuals to have access to my records.

I understand I will receive a copy of the patient information sheet and the informed consent form.   

___________________________ __________________

Signature / Thumb Impression of subject Date of signature

______________________________

Printed name of the subject in capitals

_______________________________________ __________________

Signature of the person conducting the Date of Signature

informed consent discussion

Dr. Himanshu

________________________________________ __________________

Signature of impartial witness Date of signature

________________________________________

Printed name of the impartial witness in capitals

Appendix 2

Study Proforma 

Serial number

Name

Age

Sex

Occupation

Address

Contact No.

ID No.

Weight (In Kg)

DOA

DOO

DOD

Diagnosis

HISTORY OF ILLNESS:

EXAMINATION FINDINGS: General examination

Conscious level

Built

Pallor

Vitals

LOCAL EXAMINATION: 

                                                          Left                                   Right

                a. Tenderness

                b. Deformity

FFD (fixed flexion deformity)

Varus\Valgus

                c. Knee ROM

                d. Distal Neurovascular status

INVESTIGATIONS:

      X-ray film of the concerned knee joint AP & True Lateral view

      X-Ray Scanogram of the concerned lower limb.

PREOPERATIVE DATA: 

                                                               Left       Right

Preoperative ROM

FFD

Valgus/varus

Mechanical alignment angle on scanogram 

Knee Society Score

INTRAOPERATIVE DATA:

Alignment assessment with alignment rod

                   Desired alignment achieved.

                   Desired alignment could not be achieved.

    POSTOPERATIVE DATA:

                                                    Group 1                             Group 2 

FOLLOW-UP:

At 2 weeks

Knee ROM

Knee society score

At 6 weeks

Knee ROM

Knee society score

At 3 months

Knee ROM

Knee society score

At 6 months

Knee ROM

Knee Society Score
